# Application of Additive Layer Manufacturing Technique on the Development of High Sensitive Fiber Bragg Grating Temperature Sensors

**DOI:** 10.3390/s18124120

**Published:** 2018-11-24

**Authors:** Arnaldo Leal-Junior, Jonathan Casas, Carlos Marques, Maria José Pontes, Anselmo Frizera

**Affiliations:** 1 Graduate Program of Electrical Engineering, Federal University of Espirito Santo, Vitoria 29075-910, Brazil; mjpontes@ele.ufes.br (M.J.P.); frizera@ieee.org (A.F.); 2 Biomedical Engineering, Escuela Colombiana de Ingeniería Julio Garavito, Bogotá 111166, Colombia; jonathan.casas@escuelaing.edu.co; 3 Instituto de Telecomunicações and Physics Department & I3N, University of Aveiro, Aveiro 3810-193, Portugal; carlos.marques@ua.pt

**Keywords:** fiber Bragg gratings, temperature sensor, additive layer manufacturing, 3D printing

## Abstract

This paper presents the development of temperature sensors based on fiber Bragg gratings (FBGs) embedded in 3D-printed structures made of different materials, namely polylatic acid (PLA) and thermoplastic polyurethane (TPU). A numerical analysis of the material behavior and its interaction with the FBG sensor was performed through the finite element method. A simple, fast and prone to automation process is presented for the FBG embedment in both PLA and TPU structures. The temperature tests were made using both PLA- and TPU-embedded FBGs as well as an unembedded FBG as reference. Results show an outstanding temperature sensitivity of 139 pm/°C for the FBG-embedded PLA structure, which is one of the highest temperature sensitivities reported for FBG-based temperature sensors in silica fibers. The sensor also shows almost negligible hysteresis (highest hysteresis below 0.5%). In addition, both PLA- and TPU-embedded structures present high linearity and response time below 2 s. The results presented in this work not only demonstrate the feasibility of developing fully embedded temperature sensors with high resolution and in compliance with soft robot application requirements, but also show that the FBG embedment in such structures is capable of enhancing the sensor performance.

## 1. Introduction

The use of flexible or soft structures on the development of actuators, robots and devices is an emerging trend in the last few years [1]. The so-called soft robotics involve the use of flexible fluidic actuators, shape memory materials, and electro active polymers as actuators in conjunction with rubbers, plastics and flexible cables, which result in a flexible robot [2]. These advantages are especially desirable in the development of wearable robots, where the robot can be optimally designed and controlled for each user, achieving the so-called human-in-the-loop design [3]. Soft robotics also find numerous applications in the biomedical field, which include wearable robots, prostheses, surgical and assistive devices due to the biocompatibility and biomimicry of such soft materials that are also employed as artificial organs as well as in body simulations, as summarized in [4]. For the industrial environment, soft robots can be used to perform activities that the conventional rigid robots cannot, such as grasping fragile objects. In addition, due to their higher flexibility, such robots can be regarded as a safer solution in human–robot interaction factories or workplaces [5].

3D printing technology is an additive layer manufacturing (ALM) process, generally made by fused deposition modeling (FDM) in which hot or melted polymers are injected layer-upon-layer to form the desired structure [6]. This technology has enabled the development of numerous flexible structures in soft robotics (summarized in [7]) due to its advantages of relative low cost, flexibility in design, high repeatability and no need for performing additional post-fabrication process (such as sanding, milling and scraping) [8]. Thus, many soft robotics applications rely on 3D-printed structures, which can be created using different materials such as acrylonitrile butadiene styrene (ABS), polycarbonate (PC) and polylatic acid (PLA) as well as materials with higher flexibility than the former, such as thermoplastic polyurethane (TPU). 

For accurate control of any robotic device, the sensor’s system plays a crucial role on the accuracy of control, which enable the robots to perform highly precise and demanding tasks such as in robotic assisted surgeries and human–robot interaction [5]. In these cases, the conventional electronic sensors can suffer from lack of robustness, difficult installation and the flexibility requirements can inhibit the application of several electronic sensors [9]. In addition, the electromagnetic field sensitivity of electronic sensors can harm their applications on the actuators instrumentation, which generally employs electric actuators being activated constantly [10]. Furthermore, for invasive biomedical applications, e.g. drug delivery and surgical devices, the biocompatibility constraints of the whole system, including the sensors, can limit the application of most commercially available sensors [11]. 

Optical fiber sensors represent a continuously growing research field in both photonics and sensors communities [12]. Advantages such as compactness, electromagnetic fields immunity, passive operation, multiplexing capabilities, chemical stability and biocompatibility [13] have led to the widespread of this sensing technology. Many applications of optical fiber sensors are proposed including industrial [14], structural health monitoring [15], biochemical [16] and medical [17] applications. Optical fiber sensors also offer the possibility of using many different approaches for the sensor operation, which include intensity variation [18], long period gratings [19], fiber Bragg gratings (FBGs) [20], non-uniform gratings [21], nonlinear effects [22] and interferometers [23]. Among the aforementioned types of optical fiber sensors, FBGs present high multiplexing capabilities, where quasi-distributed sensor arrays can be obtained with high spatial resolution. These attractive features of FBGs have enabled the development of FBG-based sensors for temperature [24], strain [25], liquid level [26], pressure [27], force [28] and torque monitoring [29]. In addition, the FBG advantages are well-aligned with all the requirements of soft robotics and their applications on soft robotics bring important features and benefits to robotic devices based on flexible structures and actuators.

Another important advantage of optical fiber sensors is their ability to be embedded in different structures (rigid and flexible). Thus, FBG sensors are already embedded in 3D-printed structures [30,31,32] and it is possible to foresee the 3D printing technology as the link between the FBG-based sensors and the soft robotics devices. In this way, the soft robotic device can be fabricated using 3D printing (as previously shown in [7]) with an embedded FBG-based sensor system for measuring different parameters. Furthermore, the benefits of embedding FBG sensors in 3D-printed structures are already demonstrated, where it is possible to obtain a sensor that can withstand forces as high as 1 kN with enhanced temperature sensitivity [32]. In addition, Homa et al. [33] presented the spectral characteristics of FBGs embedded in PLA structures under different strain and temperature conditions.

Since the flexible structures and actuators in soft robotics are generally made of polymers, temperature plays an important role in the robot’s operation due to the polymer viscoelastic nature. The polymer viscoelasticity leads to a non-constant response to stress and strain [34], in which the polymer’s Young’s modulus varies due to temperature deviations [35]. For this reason, temperature monitoring can compensate these effects on the actuator structure, which enables an accurate and reliable sensor system. In addition, environmental temperature monitoring is important in biomedical, industrial and structural health monitoring applications [13], which are application fields for soft robotics devices [5].

Aiming at developing a fully embedded temperature sensor with high resolution and in compliance with soft robot application requirements, this paper presents the design of FBG-embedded, 3D-printed temperature sensors. To verify the sensor behavior in different materials, the FBG was embedded in 3D-printed structures made of PLA and TPU, where the sensitivity, linearity and hysteresis of each sensor were compared with each other and an unembedded FBG sensor used as reference. The proposed temperature sensor has a compact design, can be easily fabricated with commercially available 3D printers, has electromagnetic field immunity and can be readily employed on the next generation of soft robotics. Moreover, none of the aforementioned works [32,33,36] present a thorough study on the sensor hysteresis, linearity and response times, as the one proposed in this work. Additionally, to the authors’ best knowledge, this is the first FBG embedded in a flexible 3D-printed structure as the one proposed in TPU. The comparison of this material with other 3D-printed structures brings important knowledge for the design of flexible structures with embedded sensors, which also complements the work presented in [32], where the infill density was evaluated, but for only one material (ABS in that case). It is also noteworthy that the temperature sensor proposed in this work also has much higher sensitivity (with negligible hysteresis) than the ones previously reported using similar methods. 

This paper is divided as follows. Section 1 presented the motivation and introductory aspects of the work. Section 2 describes the operation principle of the sensor with analytical and numerical approaches. Section 3 depicts the experimental setup used in the sensor characterizations. Results and discussions are presented in Section 4. Finally, the concluding remarks and future investigations are discussed in Section 5.

## 2. Operation Principle and Numerical Simulations

The proposed temperature sensor is based on FBGs embedded in a 3D-printed structure. FBGs are created through the fiber exposure on a periodic intensity pattern, which results in a refractive index modulation [37]. There are different ways to create such modulation: interference between two beams [37], phase mask [20] and direct writing using a femtosecond laser [38]. The Bragg wavelength (*λ_B_*) is directly related to the effective refractive index (refractive index with the modulation created by the intensity pattern) and the grating period as follows:

(1) $$\lambda_{B}=2n_{eff}\Lambda ,$$

where *n_eff_* is the effective refractive index and *Λ* is the grating period. Thus, variations in the grating period and refractive index lead to a shift of the Bragg wavelength. The grating period changes with variations in the fiber length, which can be created through axial strain and thermal expansion, whereas the refractive index of a fiber also varies with temperature and strain through the thermo-optic and photoelastic effects, respectively. Therefore, a FBG is intrinsically sensitive to temperature and strain following Equation (2).

(2) $$\Delta \lambda_{B}=\left[(1-P_{e})\varepsilon_{fiber}+(\alpha +\zeta )\Delta T\right]\lambda_{B}.$$

In Equation (2), *ε_fiber_* is the strain on the fiber, *P_e_* is the photoelastic constant, α is the fiber’s thermal expansion coefficient, *ζ* is the thermo-optic coefficient, and Δ*T* is the temperature variation. In this way, if the fiber is submitted to only temperature variations (without strain), the wavelength shift (*λ_B_*) is related to the initial Bragg wavelength, thermo-optic and thermal expansion coefficients, where typical values for FBG inscribed in silica fibers are 8–11 pm/°C [39]. 

It is important to note that, if the fiber is embedded in a material, the temperature behavior of the material must be considered. In this work, we considered two polymers commonly used as filaments of 3D printers: PLA and TPU. The employed polymers have different thermal expansion coefficients as well as mechanical properties. In both cases, the FBG was embedded in the center of a cylinder with 10 mm diameter and 25 mm length to guarantee that the FBG is fully embedded in the 3D-printed structure. The FBG embedment leads to a slight decrease in the reflectivity and to a red-shift on the Bragg wavelength due to the strain induced by the coating material, as previously shown both numerically and experimentally in [32]. 

**Figure 1:**
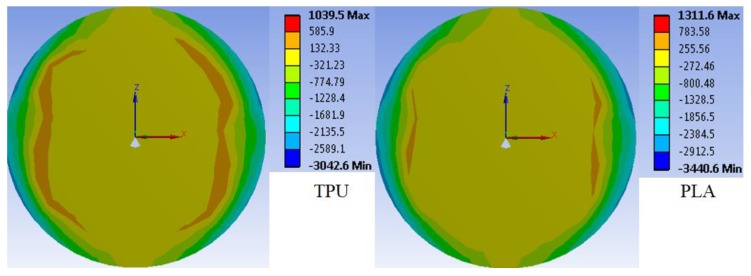
Directional heat flux simulation (in W/m^2^) of TPU and PLA structures, where the cylinders have 10 mm diameter and 25 mm length for an ambient temperature of 70 °C.

To show the temperature behavior of each polymer used on the FBG embedment, a numerical simulation using the finite element method (FEM) was performed using the software Ansys Workbench 15.0 to show the behavior of each material under temperature. The simulations were performed considering an initial temperature of 20 °C, final temperature of 70 °C and a convection coefficient of 2000 W/(m^2^ °C), which is the one considering forced convection [40]. The results for PLA and TPU are shown in Figure 1, where the directional heat flux is presented as a function of the cylinder front view. 

In Figure 1, the colormap shows a positive heat flux in the regions with color green (or higher wavelengths, such as red), whereas a negative heat flux is shown in the light green region (or lower wavelengths, such as blue). As shown in Figure 1, there is a heat flux towards the center of the cylinder for both polymers, following the fundamental law of thermodynamics, where the heat flows from the hotter environment (the ambient at 70 °C in this case) to the colder body region (center of the cylinder). Comparing the values of heat flux for both polymers, the PLA shows higher values of heat flux, which can indicate that the PLA reaches the steady state faster than TPU, leading to a lower response time for the temperature sensor. However, the difference between the structures is not high, which can also indicate that temperature sensors with TPU and PLA will present similar values of response time.

**Figure 2:**
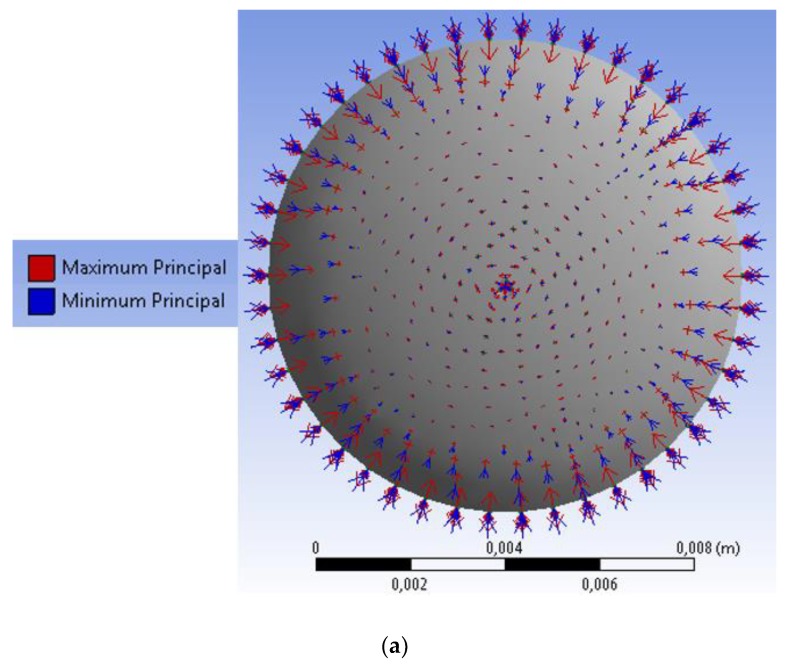

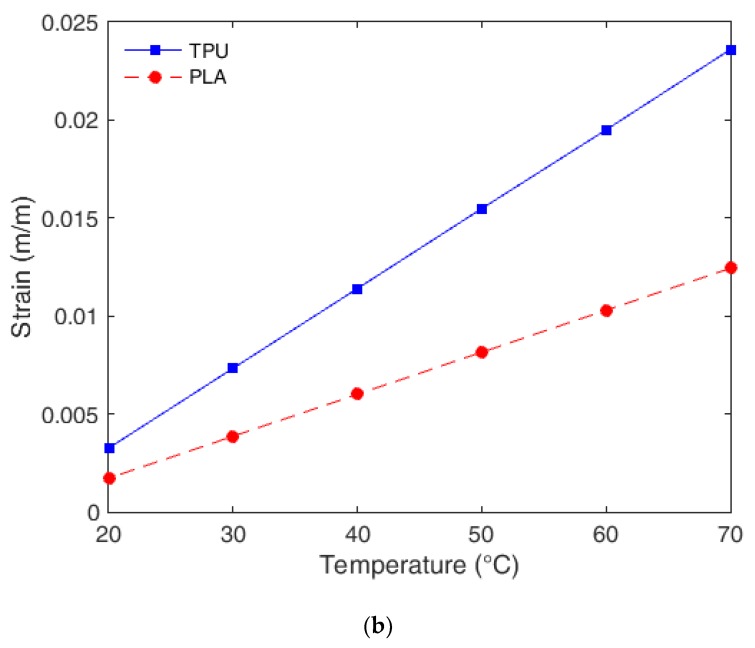
(**a**) Principal strain vector for the TPU structure. (**b**) Strain on the cylinder center for TPU and PLA as a function of the temperature.

The temperature variation on the coating materials leads to a thermal expansion of the polymers, thus, from the results in Figure 1, it is possible to infer that such thermal expansion will create a strain vector pointing towards the center of the cylinder, i.e., the region where the FBG is embedded. To verify this assumption, another simulation was performed. In this case, the analysis was made with respect to the vector of the principal strain (as shown in Figure 2a for the TPU case). It is worth mentioning that the same behavior occurs when the simulation was performed with PLA. In addition, in this simulation, we also considered a V-groove in the center of the cylinder, which is the region where the FBG was positioned. Thus, when the temperature increases, the FBG is also subjected to a strain from the thermal expansion of the 3D-printed structure. To estimate the strain which the FBG would be subjected, another simulation was performed, where the strain (as a function of the temperature) in the vicinity of the FBG for both PLA and TPU are presented in Figure 2b. The differences between the proposed sensors and the previous ones presented in the literature (e.g., [32]) are related not only to the material used in FBG embedment, but also the sensor structure. As shown in Figure 2a, the sensor was embedded in a cylindrical structure with 10 mm diameter (in contrast with [32], where a rectangular shape was used). This structure leads to a uniform distribution of the thermally induced strain on the structure, which can reduce the sensor hysteresis. In addition, this high diameter of the structure is also responsible for a higher strain transmitted to the FBG due to the polymer’s thermal expansion, resulting in higher temperature sensitivity when compared with previously reported solutions. Regarding the material, we also proposed an embedment in a TPU structure, which has a Young’s modulus of about 1.5 GPa [41], three times lower than the one of PLA (3.5 GPa). It is noteworthy that the Young’s modulus of PLA is also higher than the one of ABS (2.5 GPa) [42]. If polymers are analyzed, the viscoelastic nature of such materials must be considered, as they not have constant response with stress or strain and may also present hysteresis [34]. The viscoelasticity is defined as the combination of viscous and elastic behavior of polymers. In the viscous behavior, it is expected that the polymer has a viscous-like behavior, otherwise there is an elastic-like behavior [43]. It is possible to estimate if a polymer will have a viscous or elastic behavior with its glass transition temperature (T_g_). Above this temperatures, the polymer behavior tends to be more viscous [43], which can result in higher hysteresis. Since the T_g_ of TPU is −50 °C, whereas the one of the PLA is about 120 °C, one can expect a higher hysteresis of the TPU structure, as the tested temperatures are above the material T_g_. Thus, the analytical and numerical analyses indicate that the proposed sensor approach can enhance the sensors performance, which defines the novelty of this work, where, by performing an analytical and numerical analysis of the sensor design, structure and materials used on the embedment can lead to major improvements in the sensors’ performance.

The strain in the center of the cylinder shown in Figure 2b for TPU and PLA shows a higher strain from the TPU material, which is also related to the thermal stress defined as the product of the material Young’s modulus, thermal expansion coefficient and temperature variation. Even though PLA has higher thermal expansion coefficient (4.1 × 10^−5^ °C^−1^) than TPU (2.0 × 10^−5^ °C^−1^), the Young’s modulus of the TPU is only 0.25 GPa, whereas the one of PLA is 2.30 GPa. In addition, the Poisson’s ratios of TPU and PLA are 0.49 and 0.33, respectively. Thus, considering the thermal effects in a three-dimensional body, as shown in Equation (3) for the *y* plane, TPU will present higher strains than the PLA material.

(3) $$\varepsilon_{yy}=\frac{1}{E}\left(-\upsilon \sigma_{xx}+\sigma_{yy}-\upsilon \sigma_{zz}\right)+\alpha \Delta T,$$

where *E* is the material Young’s modulus, *υ* is the Poisson’s ratio and *σ* is the stress.

Following the strain simulations, analysis of the strain on the FBG was performed. It is noteworthy that the Young’s modulus of silica (about 70 GPa) is an order of magnitude higher than the ones of TPU and PLA. Hence, one can assume that the silica will impose a restriction on the 3D-printed structure deformation proportional to the ratio between silica Young’s modulus and 3D-printed structure material Young’s modulus, as also described in [44] for a similar case. Since the PLA Young’s modulus is about ten times higher than the one of TPU, it is expected that the PLA structure will lead to the highest temperature sensitivity (due to the higher transmitted strain than TPU), even though the TPU structure shows higher strain variation with temperature. In addition, as already discussed, it is expected a lower response time from the PLA structure due to its temperature response shown in Figure 1. Following this same assumption, it is also expected that the PLA structure will present higher temperature sensitivity than the one reported in previous works with ABS structures [32]. 

## 3. Experimental Setup

To verify the assumptions presented in Section 2 and to validate the temperature sensors, experimental tests are performed with the FBG sensors embedded in both materials (TPU and PLA). An unembedded FBG is also tested to provide a comparison between all different approaches. In all tested sensors, the FBGs were inscribed using the phase mask technique with a KrF Excimer laser operating at 248 nm and present central wavelength in the C-band with a physical length of 10 mm.

**Figure 3:**
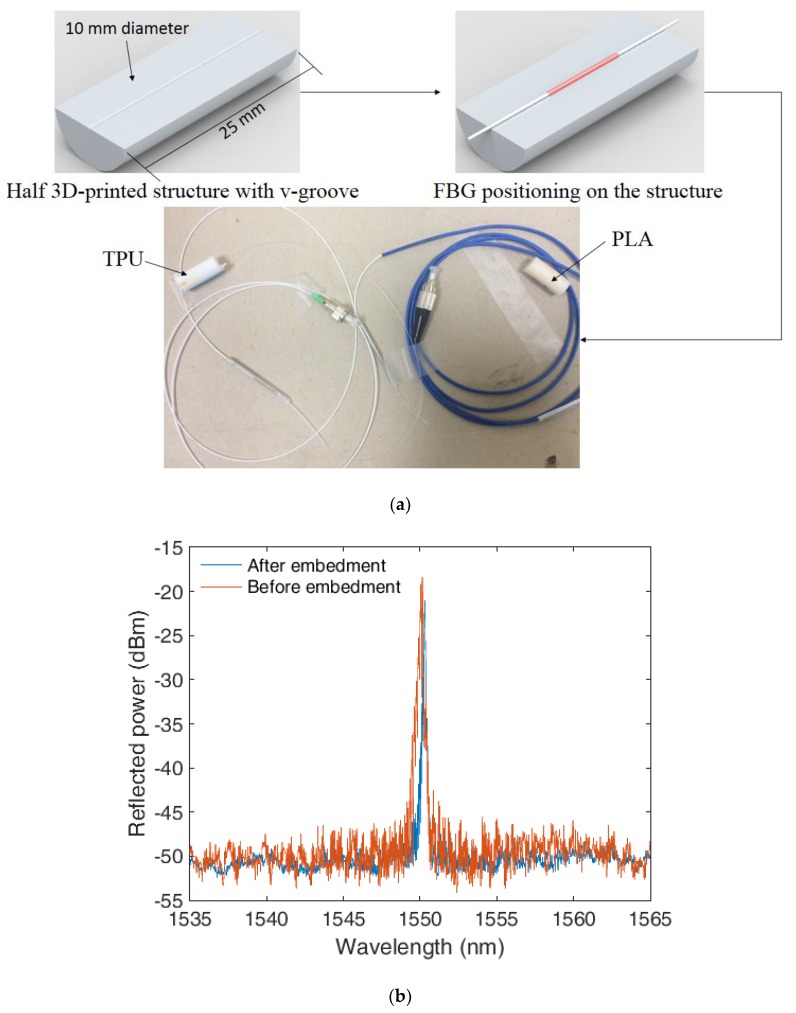
(**a**) Fabrication steps of the FBG-embedded temperature sensor and a photograph of the assembled sensor with PLA and TPU. (**b**) FBG spectra before and after embedding in the TPU structure.

For the 3D-printed structures fabrication, we used the 3D printer Sethi3D S3 (Sethi, Brazil), where all structures have a 99% infill density, which is the one that results in highest temperature sensitivity and can withstand higher forces (up to 1 kN), as discussed in [32]. As shown in Figure 3a, the 3D-printed structure has a v-groove for the fiber positioning. The embedment process comprised of pausing the printing when the v-groove was printed and, then, positioning the fiber on the v-groove with the FBG region in the center of the 3D-printed structure. Thereafter, the printing process was resumed and the FBG-embedded 3D-printed structure was fabricated. The steps for the FBG-embedded 3D-printed temperature sensor fabrication are depicted in Figure 3a, where the process of printing and embedding the fiber on the 3D-printed structure took only about 15 min. Furthermore, there is also the possibility of process automation, which leads to lower production times with the potential of large-scale production. Furthermore, FBG spectra before and after embedded in the TPU flexible structure is shown in Figure 3b, which shows a slight decrease on the reflectivity and a red-shift of about 0.1 nm. Similar results are reported in [32] for rigid materials, such as PLA. 

Following the sensor fabrication, all three samples (PLA-embedded, TPU-embedded and unembedded) temperature sensors were placed inside the thermostatic bath ECO-RE630 (LAUDA, Germany) with closed loop temperature control (temperature accuracy of 0.01 °C), where water was used as bath fluid with forced circulation. In addition, the wavelength shift of each sensor was monitored by the FBG interrogator sm125 (Micron Optics, Atlanta, CA, USA) with 1 pm wavelength resolution.

The validation tests comprised of increasing the temperature from 20 °C to 70 °C in steps of 10 °C, where each temperature was kept constant for about 5 min. Then, the temperature was decreased from 70 °C to 20 °C with the same steps to evaluate the sensor hysteresis. The response of each sensor was evaluated and compared with respect to the sensitivity, linearity and hysteresis. In addition, we also analyzed the response time of each sensor. The sensitivity was defined as the ratio between the wavelength shift and temperature variation, whereas the linearity was the determination coefficient (R^2^) between the sensor response and a linear regression curve [45]. In addition, hysteresis was the difference in the sensor response between temperature cycles (with temperature increase and decrease).

## 4. Results and Discussion

**Figure 4:**
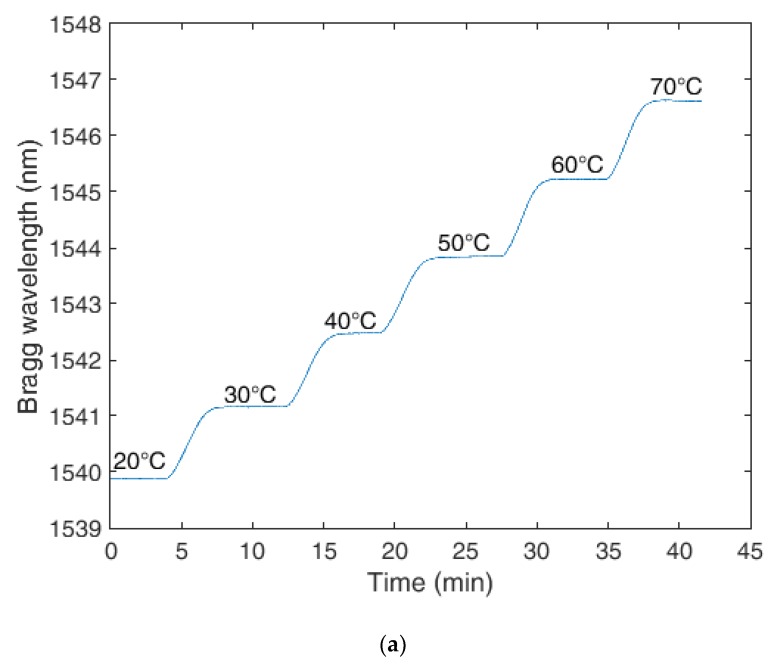

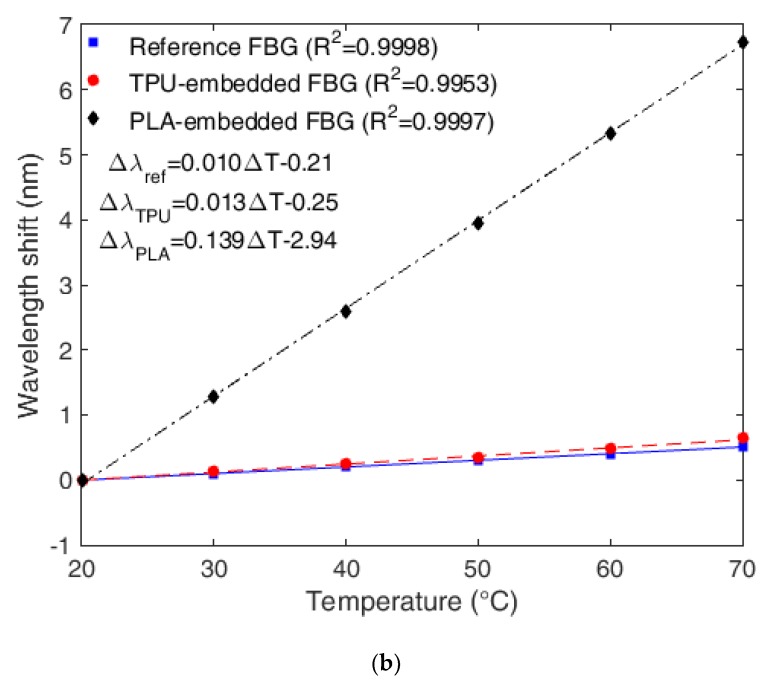
(**a**) Temperature response of the FBG-embedded 3D-printed PLA structure. (**b**) Wavelength shift as a function of the temperature for the unembedded, and TPU- and PLA-embedded FBGs.

The results for the temperature characterization (made with the experimental setup described in Section 3) are presented in Figure 4. Figure 4a shows the Bragg wavelength for the FBG embedded in the PLA structure as a function of the time. The comparison between the responses of each structure is presented in Figure 4b, where the wavelength shift of each sensor is presented as a function of the temperature. In addition, the sensitivity and linearity of each sensor are presented.

When the linearity of each sensor was analyzed, it was possible to note that, even though all sensors presented high linearity (R^2^ higher than 0.99), the highest linearity was achieved with the unembedded FBG, whereas the lowest one was found for the FBG embedded in the TPU structure. The reason for this behavior may be related to the material stiffness, which can isolate the sensor from perturbations caused by the fluid circulation inside the thermostatic bath. Thus, such effect was lower for the unembedded silica fiber due to its higher stiffness, followed by the PLA structure, which has higher stiffness than the TPU. This behavior can also be explained by the material anisotropy, which can create a non-uniform strain field on the grating when the temperature increases. For this reason, it is expected that a material with higher anisotropy will result in lower linearity for the FBG-embedded temperature sensor.

Comparing the results presented in Figure 4b, the FBG embedded in the 3D-printed PLA structure shows far higher sensitivity than the other sensors analyzed (TPU structure and unembedded). In fact, the FBG embedded PLA structure has a temperature sensitivity of about 139 pm/°C, which is one of the highest reported temperature sensitivities for silica FBG-based temperature sensors. The unembedded FBG shows a temperature sensitivity of about 10.5 pm/°C, which is in accordance with previously published works in the literature [13]. The FBG embedded in TPU structure shows a slight increase in temperature sensitivity, which is about 13.8 pm/°C. This sensitivity is higher than the theoretical limitation of the FBG temperature sensitivity (in silica fibers), which is the application of Equation (2) without strain. Thus, the theoretical limitation of temperature sensitivity for FBGs depends on the silica thermal expansion coefficient (*α* = 0.55 × 10^−6^ °C^−1^) and thermo-optic coefficient (*ζ* = 6.8 × 10^−6^ °C^−1^) as well as the Bragg wavelength. Considering the aforementioned values, the theoretical temperature sensitivity of FBGs in silica fibers is about 10.9 pm/°C. This higher temperature sensitivity of the FBG-embedded TPU structure indicates that the wavelength shift is due to not only the temperature effects in the fiber, but also to the thermally induced stress/strain of the TPU structure on the grating. The same principle occurs with the FBG embedded in the PLA structure, where more strain is transmitted to the grating due to the PLA higher Young’s modulus, which is about ten times higher when compared to the one of TPU. Interestingly, the temperature sensitivity of the PLA structure is also about ten times higher than that of the TPU, which indicates, as assumed in Section 2, the strain transmission from the 3D-printed structure to the FBG follows a direct proportionality with the materials Young’s modulus. Thus, even though the TPU structure shows the higher strain with temperature variation (see Figure 2b), only a small part of this strain is actually transmitted to the silica fiber due to its much higher Young’s modulus, whereas the strain transmission of the PLA structure is higher, resulting in a higher temperature sensitivity.

**Figure 5:**
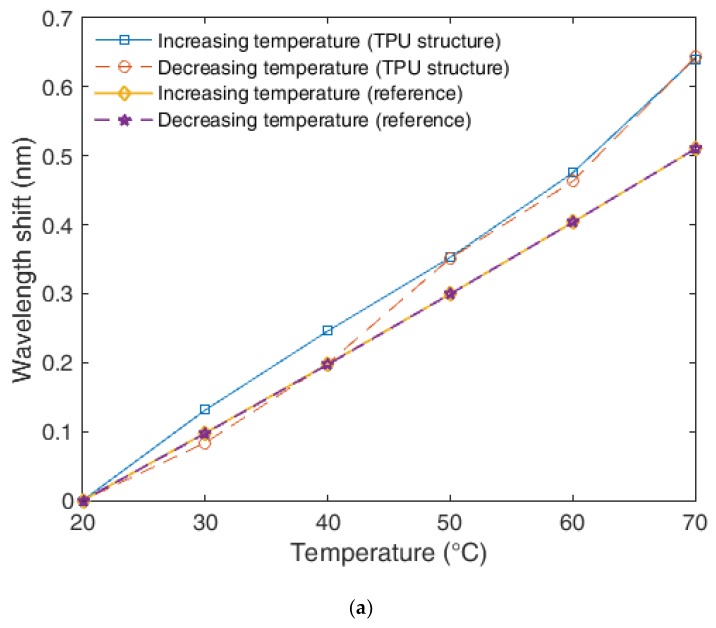

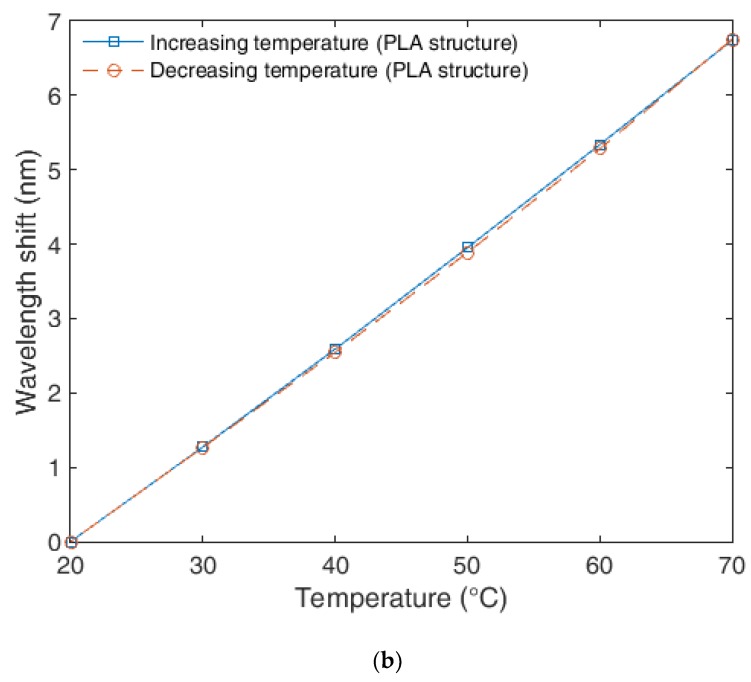
(**a**) Unembedded and FBG-embedded 3D-printed TPU structure response for temperature cycle. (**b**) FBG-embedded 3D-printed PLA structure response for temperature cycle.

For the hysteresis analysis, two temperature cycles were performed, where the temperature was increased from 20 °C to 70 °C and, then, decreased from 70 °C to 20 °C. The results of this test are presented in Figure 5. To provide a better visualization of each curve, Figure 5a depicts the results obtained in the temperature cycles for the FBG-embedded TPU structure and unembedded FBG, whereas Figure 5b shows the response for the FBG-embedded PLA structure due to the large sensitivity difference between the 3D-printed structures.

The comparison between all three samples shows a lower hysteresis for the unembedded FBG, where a negligible hysteresis was obtained (below the FBG interrogator resolution). In contrast, a much higher hysteresis was found in the TPU structure, where a hysteresis as high as 5% was obtained. The hysteresis was estimated through the ratio of the highest difference between increasing and decreasing temperature with the wavelength shift obtained in the whole cycle (20 °C to 70 °C). This high hysteresis found for the TPU is related to the material response, which can present hysteresis due to its viscoelasticity [34] and the material anisotropy. Same analysis was made for the FBG-embedded PLA structure, but a much lower hysteresis was obtained. In this case, the maximum hysteresis is below 0.5%. Therefore, the PLA is a much more suitable material for the FBG embedment in temperature sensors applications, since it provides ten times higher sensitivity, higher linearity and much lower hysteresis than the TPU. 

**Figure 6:**
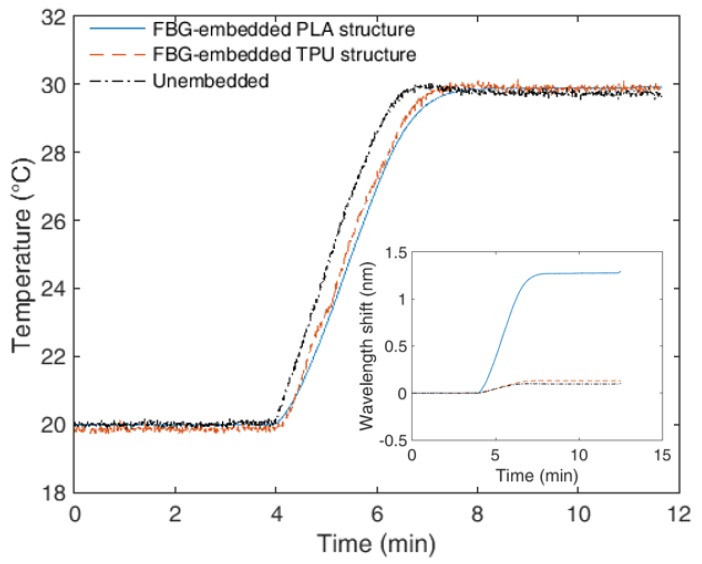
Response time of embedded and unembedded FBG sensors to a 10 °C temperature step. Inset shows the wavelength shift of each FBG as a function of the time.

The last performed analysis is the characterization of the sensor response time. In this case, a 10-°C step was applied and the deviation between the sensor response and the temperature rise was analyzed for each tested sample on the interval between 20 °C and 30 °C due to the higher stability of the thermostatic bath in this interval. To provide a better comparison between the sensors, the response of each sensor is shown with respect to the estimated temperature by applying the characterization equations obtained for each FBG sensor in Figure 4b. The response time of each sensor is depicted in Figure 6, where the response time of each sensor is the difference between the temperature rise time obtained from each sensor with the temperature increase time of the thermostatic bath. Thus, it is possible to observe the transient response of each sensor, where the unembedded FBG presented the highest slope on the temperature curve (as a function of time), indicating its lower response time, i.e., it reaches the steady state temperature faster than the other tested sensors. The response time of the thermostatic bath is about 140 s, i.e., the thermostatic bath takes 140 s to increase the temperature from 20 °C to 30 °C. In addition, Figure 6 inset depicts the high sensitivity difference between the sensors at this temperature range.

The response time of each sensor as a function of the temperature was obtained by subtracting the time that each sensor takes to increase the temperature from 20 °C to 30 °C with the time that the thermostatic bath takes to increase the temperature in this same range. Thus, this analysis already accounts for the dynamics of the thermostatic bath (2.8 °C/min), which is limited to the bath fluid (water) dynamics, harming the application of higher temperature rates. Nevertheless, analyzing a lower temperature rate (0.8 °C/min), similar response times were found. The unembedded FBG shows the lowest response time, since it only takes 0.3 s to respond a temperature variation of 1 °C. For the 3D-printed structures, the response time is higher, which is expected due to the higher thermal resistance imposed by both PLA and TPU structures. The response times in these cases are 1.6 s and 1.8 s for the TPU and PLA, respectively. The maximum response time variation, when compared with the case where the lower temperature variation rate was applied (0.8 °C/min), was below 3% (for the TPU-embedded FBG). Such lower variation on the response time indicates that the response times estimated with the employed method are not influenced by the thermostatic bath dynamics (since these dynamics are compensated for in the response time calculation). The reason for such lower response time for the TPU structure is related to its higher thermal conductivity (1.9 W/(m °C)) when compared with the one of PLA (1.3 W/(m °C)). Following Fourier’s law, such higher thermal conductivity coefficient leads to a faster temperature response. 

To summarize the analyses performed, Table 1 shows a comparison between the sensors with respect to linearity, sensitivity, hysteresis and response time. In addition, we also compares the temperature resolution of each sensor, which was estimated considering the sensitivity of each sensor and the FBG interrogator wavelength resolution (1 pm). Thus, it is expected a higher resolution of the PLA-embedded FBG due to its higher sensitivity. The results presented in Table 1 show that a PLA-embedded FBG sensor is a suitable solution when high precision, sensitivity and resolution is required and the 3D printing methods employed for its fabrication are well-aligned with the requirements for soft robotics applications. Thus, for temperature sensing applications, the PLA is the preferred material for the FBG embedment, since this material provides higher sensitivity and linearity with lower hysteresis than the other two tested options. The thermally induced strain sensitivity of each material for FBG embedment depicted in Figure 2b is also shown in Table 1, since it indicates that the TPU structure may present some advantages in strain sensing applications due to its higher flexibility when compared with the PLA.

## 5. Conclusions

This paper presents the development of a FBG-embedded temperature sensor using ALM techniques, specifically, fused deposition modeling, the so-called 3D printing, where the FBG was embedded in 3D-printed structures made of two different materials, namely PLA and TPU. A numerical analysis of the material behavior under temperature and its effects on the FBG was performed prior to the sample fabrication using the finite element method. Thereafter, the FBG-embedded 3D-printed structures were fabricated using PLA and TPU, whereas an unembedded FBG was used as reference to provide a comparison between the different sensors. The experimental analysis shows a high temperature sensitivity of the PLA-embedded FBG sensor, which is one of the highest reported sensitivities for temperature sensors (139 pm/°C). In addition, the PLA-embedded sensor shows an almost negligible hysteresis, where the maximum hysteresis was 0.5%. In addition, the TPU-embedded structure also shows higher sensitivity than the unembedded FBG and lower response time than the PLA-embedded one. The results reported in this work show the feasibility of applying ALM in conjunction with optical fiber sensors to provide a substantial enhancement on the optical fiber sensor performance. Furthermore, as the soft robotics field moves towards the use of 3D printing in actuators and flexible structures design, the proposed FBG-embedded sensor is an important tool in this application field. Thus, it is possible to design actuators, structures and sensors with the same manufacturing process, which results in a portable and highly customizable system.

## Figures and Tables

**Table 1:** Comparison between the performance parameters of embedded and unembedded FBGs.

	PLA-embedded	TPU-embedded	Unembedded
Sensitivity	139.0 pm/°C	13.8 pm/°C	10.5 pm/°C
Linearity	0.9997	0.9953	0.9998
Hysteresis	<0.5%	5%	<0.1%
Response time	1.8 s	1.6 s	0.3 s
Resolution	0.007 °C	0.07 °C	0.09 °C
Thermally induced strain	400 με/°C	214 με/°C	-
